# Effect of Holding Time on Densification, Microstructure and Selected Properties of Spark Plasma Sintered AA7075-B_4_C Composites

**DOI:** 10.3390/ma15062065

**Published:** 2022-03-11

**Authors:** Anna Wąsik, Beata Leszczyńska-Madej, Marcin Madej, Rafał Rubach, Dariusz Garbiec

**Affiliations:** 1 Faculty of Non-Ferrous Metals, AGH University of Science and Technology, 30 Mickiewicza Ave., 30-059 Krakow, Poland; anna.wasik@agh.edu.pl; 2 Faculty of Metals Engineering and Industrial Computer Science, AGH University of Science and Technology, 30 Mickiewicza Ave., 30-059 Krakow, Poland; mmadej@agh.edu.pl; 3 Łukasiewicz Research Network – Poznań Institute of Technology, 6 Ewarysta Estkowskiego St., 61-755 Poznan, Poland; rafal.rubach@pit.lukasiewicz.gov.pl (R.R.); dariusz.garbiec@pit.lukasiewicz.gov.pl (D.G.)

**Keywords:** spark plasma sintering, AA7075-B_4_C, aluminum matrix composite, microstructure, mechanical properties

## Abstract

The paper presents the effect of the holding time, varying between 1 min 15 s and 10 min, on the microstructure evolution and development of selected properties of spark plasma sintered AA7075-based composites reinforced with 3, 5 and 10 wt% sub-micro B_4_C powder. The sintering temperature and the compaction pressure were 500 °C and 80 MPa, respectively. Composites with a near full density of 96–97% were obtained. Microstructure studies were performed employing the techniques of light microscopy and scanning electron microscopy, along with an analysis of the chemical composition in micro-areas. Additionally, the phase composition was investigated by means of X-ray diffraction. In addition, hardness and flexural strength tests were performed. It was found that the holding time did not significantly influence the microstructures of the examined materials nor the hardness or flexural strength. The sintered composites had a fine-grained microstructure with a strengthening phase located at the grain boundaries. As a result of the spark plasma sintering process, fine precipitates of intermetallic phases were also observed in the aluminum grains, suggesting partial supersaturation, which occurred during fast cooling.

## 1. Introduction

Metal matrix composites reinforced with ceramic particles have found wide applications in the aerospace, electronics, and manufacturing industries due to their high strength-to-weight ratio and good wear resistance. Composites based on light metals, for example, aluminum and its alloys, deserve special attention because they are additionally characterized by low density. However, to meet the application requirements, the material must be properly strengthened. Therefore, ceramic particles are introduced into the metal matrix to improve such composite properties as hardness, strength, wear resistance, and stiffness [1]. The commonly used ceramic particles are B_4_C, SiC, TiO_2_, TiC, TiB_2_, Al_2_O_3_, WC, ZrC, ZrO_2_, and graphite [2,3,4,5]. Among them, boron carbide (B_4_C) is one of the most promising candidates for a reinforcing phase in metal matrix composites on account of its superior properties. B_4_C is one of the hardest ceramic materials (9.5+ on the Mohs scale) [6] characterized by high wear resistance (second after diamond) [7] and low density (2.52 g/cm^3^) [8]. This material is becoming increasingly popular as a raw material for the production of machining tools.

The effect of strengthening with B_4_C particles on the mechanical properties of aluminum and its alloys has been studied by many researchers. B_4_C particles as reinforcement have been widely used to fabricate materials characterized by increased strength and wear resistance [5,9,10,11,12]. Toptan et al. [9] investigated the effect of B_4_C volume fraction on wear behavior. The authors concluded that by increasing the volume fraction of B_4_C particles from 15% to 19%, the coefficient of friction and wear rates rose. They observed that the wear mechanism is a combination of adhesive, abrasive, and delamination wear. The authors of [10] reported an increase in hardness and tensile strength after additions of 7 and 9 wt% B_4_C particles to the AA6061 produced by melt stirring. Similarly, research by Mohammad et al. [5] demonstrated that introducing 15 wt% B_4_C nanoparticles into pure Al powder resulted in a growth in hardness from 33 HV to 164 HV and ultimate compressive strength from 130 MPa to 485 MPa. Karabulut et al. [11] investigated the mechanical properties of AA6061 reinforced with 5–20 wt% B_4_C particles fabricated by the powder metallurgy and hot extrusion method. They concluded that the hardness, fracture toughness, and tensile strength changed with the content of boron carbide reinforcement. The highest hardness (70 HV_3_) was noted for the composite with the addition of 20 wt% B_4_C. An opposite dependency was observed for the tensile strength; the highest value of which was reported for the addition of 5 wt% B_4_C (210 MPa). The highest fracture toughness (26 J) was exhibited by the composite with 10 wt% B_4_C. The influence of B_4_C on mechanical and tribological properties was also studied by Baradeswaran and Elaya Perumal [12], who used AA7075 as the matrix. The reinforcing particles, in the amounts of 5, 10, 15 and 20 vol.%, were introduced into the molten matrix by mechanical stirring. The authors reported that the hardness, tensile, compression and flexural strength were found to rise with an increasing B_4_C content and reached 200 HB, 300 MPa, 340 MPa and 497 MPa, respectively, measured for the addition of 20% vol.% B_4_C. A similar dependency was observed in the measured tribological properties of AA7075-B_4_C composites. The wear rate was significantly lower for the composite materials compared with the matrix. Additionally, the coefficient of friction decreased with increasing reinforcement.

The final properties of the composite material will be affected by a number of factors, ranging from the amount, particle size, morphology, and shape of the reinforcing particles, and ending with the method of manufacturing that allows uniform distribution of the reinforcement particles in the matrix. Aluminum and its alloys used as the matrix material generate an additional factor, which is a pore-free oxide layer on its surface. As aluminum is characterized by a strong affinity for oxygen, the continuous film of aluminum oxide (Al_2_O_3_) acts as a barrier to the formation of diffusion bonds between the particles during conventional sintering [13,14]. The sintering process of aluminum powders takes place at temperatures much lower than those required by aluminum oxide. Therefore, for the successful sintering of aluminum powder, the oxide film has to be interrupted. There are a few mechanisms during the sintering process that allow the alumina film to rupture and make direct contact between the particles, such as the difference in the thermal expansion coefficients between aluminum and Al_2_O_3_, thermal stresses and local melting [15].

Another factor that has to be taken into consideration is interfacial reactions that occur at the matrix/reinforcement phase boundary. Guttikonda Manohar et al. [16] reported that sintering at temperatures above 600 °C can lead to a reaction between Al and B_4_C with the possible formation of intermetallic phases such as AlB_2_, AlB_12_ and Al_3_BC. These reaction products formed at the Al/B_4_C interface have an unfavorable effect on the mechanical properties [5,17]. Y.Z. Li et. al. [18] manufactured AA6061-B_4_C composites by hot pressing at different temperatures (560 and 620 °C). The microstructure revealed reaction products (Al_3_BC and Al_12_BC_2_) of Al and B_4_C at the interface between the matrix and the reinforcement particles in hot pressed composites at an elevated temperature (620 °C). Furthermore, Zhang et al. [19] detected reaction products (AlB_2_, AlB_12_, and Al_3_BC) formed during the sintering of Al-B_4_C composites at 680 °C. The authors of [20,21] investigated, among others, the effect of various sintering techniques on the differences between intermetallic compounds in AA7075-B_4_C composites fabricated by conventional sintering (620 °C), spark plasma sintering (500 °C) and microwave-assisted sintering (500 °C). X-ray diffraction (XRD) analysis showed that the composites compacted by the conventional sintering method exhibited an intermetallic phase of AlB_2_. In the composites compacted by the spark plasma sintering (SPS) and microwave-assisted sintering (MAS) techniques, this type of phase was not detected. The same authors observed that the distribution of the reinforcement in the matrix was uniform in the composites produced by SPS and MAS, while the conventionally sintered composite exhibited agglomerations of B_4_C particles. The formation of the AlB_2_ intermetallic phase and the non-uniform distribution of reinforcement in conventionally sintered composites resulted in inferior mechanical properties. Another advantage of utilizing the SPS technique to produce composite materials over conventional powder metallurgy methods observed by the authors was a significant reduction in porosity levels. Wu et al. [22] studied the effect of plasma-activated sintering parameters on the densification and properties of AA7075-B_4_C composites. They reported that a sintering temperature of 530 °C and a holding time of 3 min were sufficient to achieve composites with near full density, characterized by high Vickers hardness (182 HV), high bending strength (1100 MPa), high compression yield strength (878 MPa) and fracture strength (469 MPa). Higher sintering temperatures and holding times reduced the properties as a result of the formation of the MgO phase.

Advanced sintering techniques such as SPS are more effective compared with traditional sintering methods owing to faster heating and a shorter holding time, and thus avoiding mass transport mechanisms that result in the formation of undesirable phases, which hinder the densification process [23,24]. Additionally, a shorter holding time allows the preservation of a finer microstructure [25]. In this technique, a pulsed DC current (Joule heating) combined with a simultaneously applied compaction pressure, contributes to the fast densification of the powders [26]. While SPS involves a lower sintering temperature than conventional techniques, spark discharges can be generated in the gaps between the powder particles, which enhance the evaporation of oxides due to a locally high temperature increase on the surface of the powder particles, mainly at the heating stage. This facilitates the neck formation necessary for effective bonding between the aluminum particles [26,27,28].

The presented study provides information on AA7075-based composites reinforced with B_4_C particles manufactured by spark plasma sintering. The aim of this study is to understand the effect of the holding time and weight content of B_4_C reinforcement on the evolution of the microstructure and development of the mechanical properties of AA7075-B_4_C composites. The novelty of this research is SPS optimization based on detailed analysis of the holding time and its effect on the compaction of the sintered compacts. Analysis of the shrinkage curves allows us to reduce the holding time as much as possible to manufacture composites with near full density. The strengths of this approach are the minimalization of energy consumption as well as the inhibition of grain growth and chemical reactions between aluminum and boron carbide.

## 2. Materials and Methods

**Figure 1:**
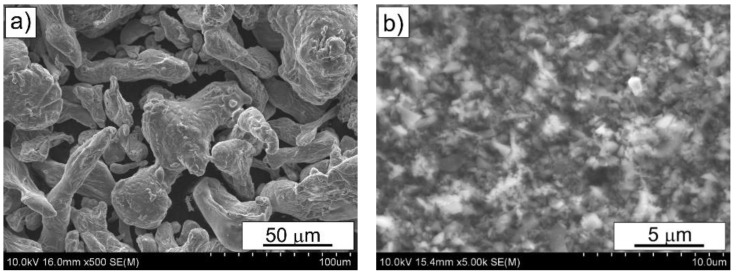
Morphology of initial powders: (**a**) Alumix 431 and (**b**) B_4_C.

In this work, an Alumix 431 powder mixture without solid lubricants (Ecka Granules, Welden, Germany) with the chemical composition shown in [29] was used as the matrix material. The powder was produced by air atomization and had an irregular shape with diameters less than 86 µm. The B_4_C powder delivered by KAMB Import-Export, Warsaw, Poland, was selected for the reinforcement phase and was obtained by powder synthesis process; the particle size was below 0.8 µm. The B_4_C particles were added in quantities of 3, 5 and 10 wt% to the matrix material. Figure 1 presents the morphology of the employed powders. 

The Alumix 431 powder was mixed with various amounts of B_4_C using a Turbula T2F (WAB, Muttenz, Switzerland) shaker–mixer for 60 min. The blended powders were initially compacted by hand into a graphite die with an inside diameter of 40 mm. The SPS process was carried out in an HP D 25/3 furnace (FCT Systeme, Rauenstein, Germany) under a vacuum of 5 × 10^−2^ mbar. The sintering temperature and compaction pressure were 500 °C and 80 MPa, respectively. The heating rate was 100 °C/min and the holding time was varied from 10 min to 1 min 15 s. The dimensions of the acquired cylindrical-sintered samples were 40 mm in diameter and 10 mm in height. In total, 6 samples were manufactured (two samples from each of the three compositions spark plasma sintered at 10 min and at the optimized time).

The relative density of the composites was measured by the Archimedes’ principle. The microstructures of the obtained composites were analyzed by means of an SU-70 (Hitachi, Tokyo, Japan) scanning electron microscope (SEM) equipped with a NORAN System 7 (Thermo Fisher Scientific, Waltham, MA, USA) X-ray microanalysis system (EDS). The phase composition was analyzed utilizing a D8 Advance (Bruker, Karlsruhe, Germany) X-ray diffractometer with Co Kα = 1.79 Å. The composite hardness was measured using the Brinell method applying a load of 31.25 kgf with a 2.5 mm diameter tungsten carbide ball indenter. The flexural test was conducted with a Z020 (Zwick Roell, Ulm, Germany) universal testing machine at a constant strain rate of 0.05 mm/s via the three-point bending test in accordance with the PN EN ISO 7438 standard. The test was carried out on bars with dimensions of 4 mm × 4 mm × 40 mm cut from sintered samples. Three specimens were tested for each variant, and the flexural strength values were averaged. The fracture surfaces from the flexural test were examined by SEM. 

## 3. Results and Discussion

**Figure 2:**
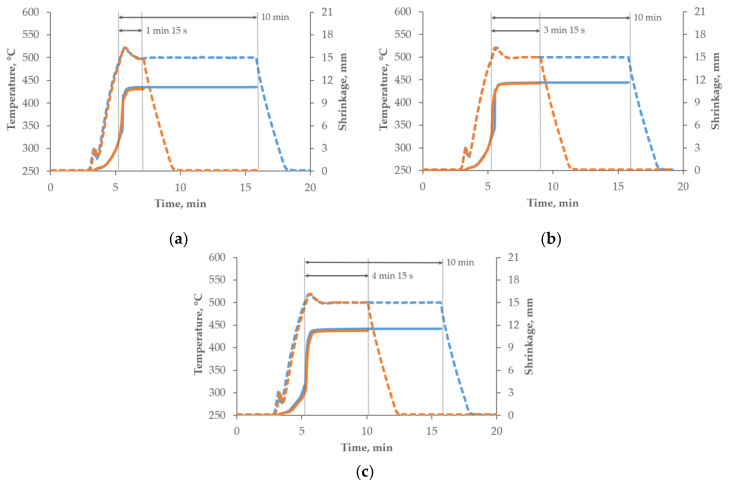
Shrinkage curves of (**a**) AA7075-3wt%B_4_C, (**b**) AA7075-5wt%B_4_C and (**c**) AA7075-10wt%B_4_C spark plasma sintered at various holding times (10 min: blue curve; optimized time: orange curve) marked by arrows (temperature: dotted curve; shrinkage: solid curve).

The internal data logger of the SPS furnace enabled in situ observation of the sintering temperature and punch displacement, measured by an inductive transducer. Analysis of the time-dependent displacement supplied preliminary information about the progress of densification (shrinkage of the compact). Typical shrinkage curves are shown in Figure 2, where it is clearly seen that densification started at approximately 300 °C, then sped up at approximately 350 °C, with significant growth when the sintering temperature of 500 °C was reached. Based on the results presented by Garbiec and Siwak [29], the first SPS cycles for all the compositions (3, 5 and 10 wt% B_4_C) started from the holding time of 10 min. A plateau in the shrinkage curves of those samples was clearly seen in all the cases after a holding time of <1 min 15 s for 3 wt% B_4_C, <3 min 15 s for 5 wt% B_4_C, and <4 min 15 s for 10 wt% B_4_C. This meant that with the applied SPS parameters, the densification was completed in times significantly less than 10 min. It allowed us to optimize the holding time of each composition, and the next SPS cycles were carried out with holding times of 1 min 15 s, 3 min 15 s and 4 min 15 s for 3 wt% of B_4_C, 5 wt% of B_4_C and 10 wt% of B_4_C, respectively. Shrinkage curves with a clearly visible plateau similar to the ones recorded from the samples sintered at 10 min were also obtained, which meant that densification was completed at the end of the holding stage. 

**Figure 3:**
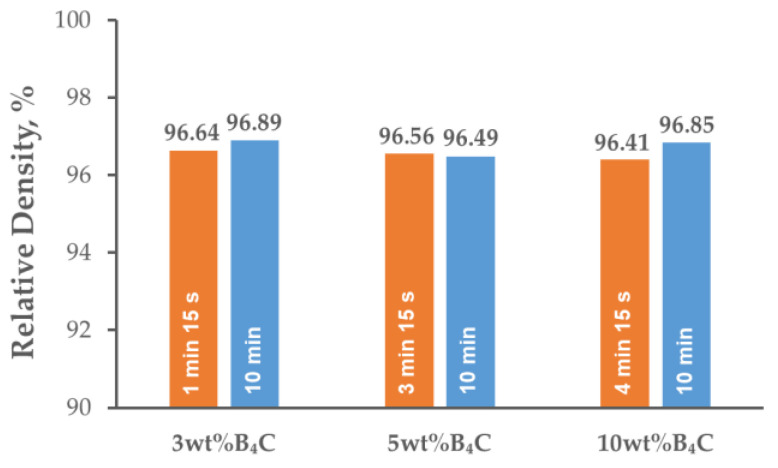
Relative densities of AA7075-*x*B_4_C (*x* = 3, 5, 10 wt%) composites depending on B_4_C content and holding times.

Figure 3 presents the relative densities, determined by the Archimedes’ method, of the AA7075-B_4_C composites sintered at various holding times. The relative density of the sintered composites reached almost 97% and remained at the same level for all the variants of the B_4_C particle weight fractions and holding times of the SPS process. The densification of the composite material was high, and a porosity of more than 3% was present mainly in the regions of the B_4_C particle clusters. When compacting a mixture of ductile matrix particles and fine, hard reinforcement particles, the main limitation in achieving full densification is the tendency of the reinforcing phase particles to form agglomerates. Although a spark discharge occurs between the individual particles of the B_4_C phase, the plastic deformation was slight because of the fact that the sintering process was carried out at a much lower temperature than the melting point of the boron carbide. Therefore, the loosely assembled particles present in the material reduced the degree of densification of the material [28]. 

**Figure 4:**
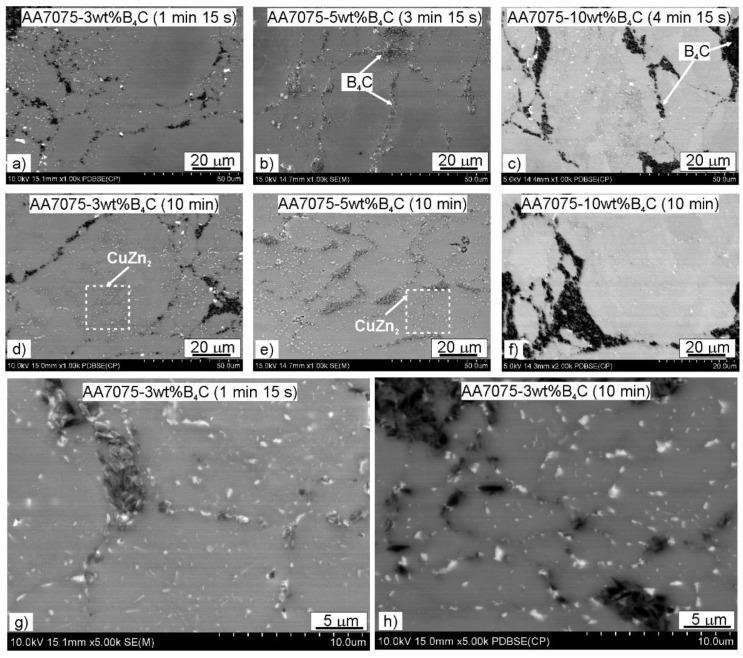
Microstructure of composites spark plasma sintered at various holding times: (**a**) AA7075-3wt%B_4_C (1 min 15 s), (**b**) AA7075-5wt%B_4_C (3 min 15 s), (**c**) AA7075-10wt%B_4_C (4 min 15 s), (**d**) AA7075-3wt%B_4_C (10 min), (**e**) AA7075-5wt%B_4_C (10 min), (**f**) AA7075-10wt%B_4_C (10 min), (**g**) AA7075-3wt%B_4_C (1 min 15 s, higher magnification), (**h**) AA7075-3wt%B_4_C (10 min, higher magnification).

**Figure 5:**
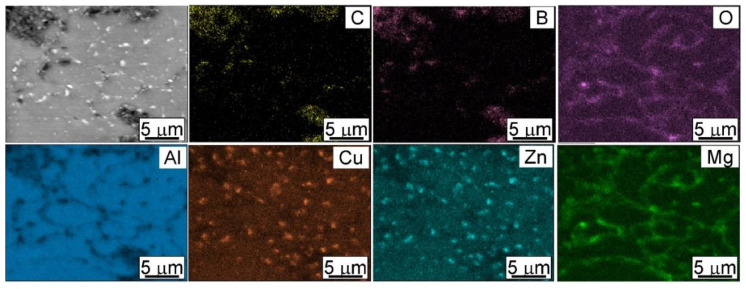
SEM and corresponding EDS mapping micrographs of AA7075-3wt%B_4_C spark plasma sintered at 10 min.

**Figure 6:**
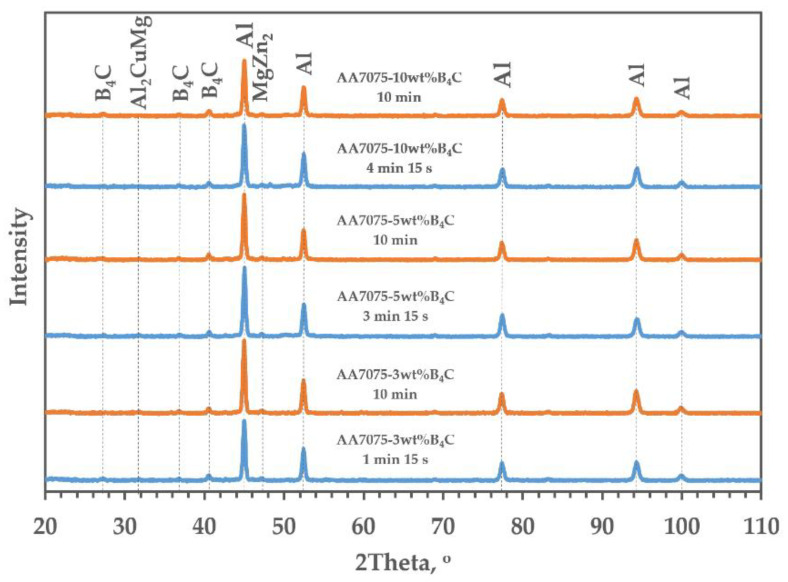
X-ray diffraction spectra of AA7075-*x*B_4_C (*x* = 3, 5, 10 wt%) depending on SPS parameters.

The microstructures of the investigated materials do not depend significantly on the holding time, as can be observed in the shrinkage curves presented in Figure 2. The temperature peak indicates that the greatest sintering effects occurred just after reaching the sintering temperature. The sintered compacts were characterized by a fine-grained microstructure. The grain size was similar to the original powder particles, on average ranging from 50–70 µm. There was no effect of the strengthening phase content or the sintering parameters on the grain size. The strengthening phase particles were distributed at the grain boundaries. There were also some precipitates visible in the microstructure (Figure 4). Based on the analysis of the chemical composition in the micro-areas and also XRD analysis, it was found that these precipitates were rich in Zn, Cu and Mg. The precipitates likely to be observed are the following phases: MgZn_2_, CuZn_2_, Al_2_CuMg (Figure 5 and Figure 6). They are probably formed in parallel with the sintering process and during cooling to ambient temperature. The precipitates present in the composites sintered for 10 min were larger and mostly had a globular shape. The reinforcing phase was found to be relatively homogeneously distributed. Nevertheless, the manufacturing process did not allow the formation of agglomerations in the reinforcing phase to be avoided, which was especially visible in the higher content of the strengthening phase, and promoted the occurrence of unfavorable porosity in the microstructure, concentrated mainly inside the clusters of this phase.

The element distribution maps confirmed, in addition to the main elements of the Alumix 431 powder matrix and the B_4_C strengthening phase, the presence of alumina. The presence of oxides is associated with the strong affinity of aluminum for oxygen. To form diffusion bonds between the powder particles, exposed, oxide-free active powder surfaces are necessary. During the SPS process of Al powder, the alumina layer on the surface of the Al particles is partially broken by the spark discharges and the applied compaction pressure.

Thus, it was observed that the application of SPS as a densification technique of the examined composite materials allowed unfavorable solid-state reactions between aluminum and B_4_C reinforcement to be avoided, with the formation of undesirable phases such as Al_4_C_3_, Al_3_BC AlB_2_, AlB_12_ and Al_3_BC. The instantaneous temperature changes resulting from the flow of pulsed current were insufficient for these reactions to take place, and after the contact surface of the particles was increased, it was already too low. Therefore, we can avoid these reactions because the SPS does not require the use of high sintering temperatures; a short holding time was also a favorable factor here (the established temperature of sintering is 500 °C).

**Figure 7:**
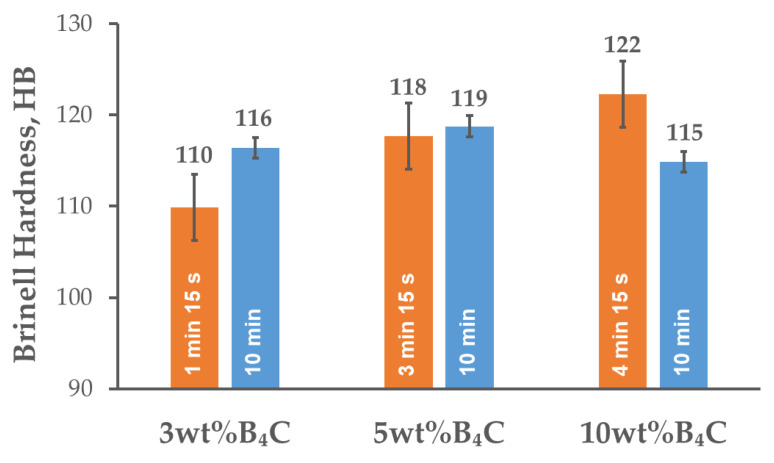
Brinell hardness of AA7075-B_4_C composites spark plasma sintered at various holding times.

Figure 7 presents the evolution of Brinell hardness in the composites as a function of the B_4_C content and the holding time. The hardness increased slightly with the addition of B_4_C particles. Nonetheless, the differences are slight and are within the error limits when taking into consideration the composites with the contents of 5 and 10 wt% B_4_C. For the lowest reinforcement content, the hardness was improved by extending the holding time. However, it is not noticeable for the composite with the content of 5 wt% B_4_C. The highest hardness was achieved by the composites with the addition of 10 wt% B_4_C (122 HB), but extension of the holding time to 10 min resulted in its decrease. The problem may be the poor binding of the B_4_C particles present in the form of clusters; the applied sintering temperature is sufficient for the matrix material, but too low for this carbide. The rise in hardness is related to the high hardness of the B_4_C particles and the microstructure of the composite material, which testify to the homogeneous distribution of the reinforcement phase in the matrix. Furthermore, the combination of hard B_4_C particles with a soft and ductile aluminum alloy matrix generates strain energy as a consequence of the thermal expansion coefficient mismatch between these phases, thus influencing a rise in dislocation density [30,31]. The B_4_C reinforcement particles also take part in load transfer from the matrix material. In the SPS process, the DC pulse phenomenon between the particles might generate spark discharges, which can successfully detach adsorbed gas and oxide films from the particle surface, thus improving the efficiency of sintering. This fact, in addition to plastic deformation and Joule heating, reduce the holding time and temperature, limiting the grain growth. The combined dislocation strengthening, load transfer, grain refinement, and effective material densification during sintering contribute to the improved properties of the final composite material [28].

**Figure 8:**
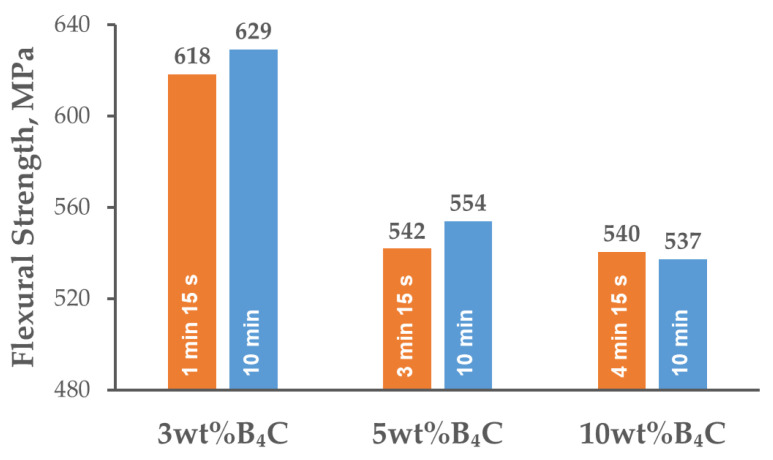
Flexural strength of AA7075-B_4_C composites spark plasma sintered at various holding times.

Completely different characteristics of the measured flexural strength values can be observed (Figure 8). The composites with 3 wt% B_4_C were characterized by the highest resistance to bending. Increasing the reinforcement content resulted in a marked decline in the flexural strength from the values of 618–629 MPa (depending on the holding time) given for a 3 wt% content of B_4_C, to 537–554 MPa for the composites with higher contents of B_4_C. The sharp edges of the hard particles can promote crack initiation during bending. A fine-grained reinforcement phase can form clusters between the larger particles of the matrix, which hindered homogeneous distribution of the reinforcing phase in the matrix during mixing in the Turbula shaker-mixer and might also have contributed to cracking. The influence of the holding time on the flexural strength was similar to that observed for the hardness measurements. For the lower contents of reinforcing phase (3 and 5 wt% B_4_C), slight growth in the flexural strength was observed with extension of the holding time. Nevertheless, for the composite with the addition of 10 wt% reinforcement, the flexural strength remained at the same level, regardless of the holding time.

**Figure 9:**
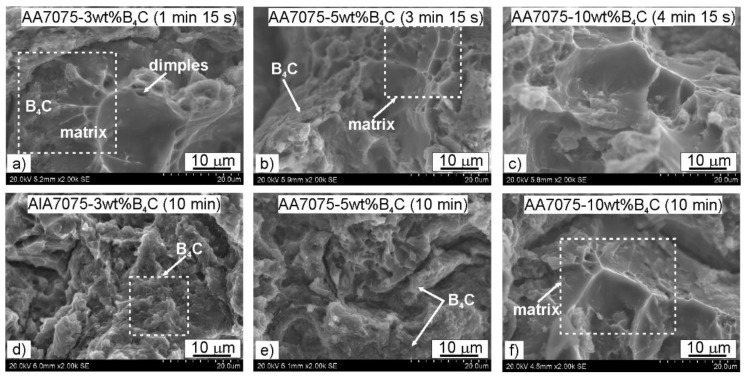
Fracture morphology of AA7075-*x*B_4_C (*x* = 3, 5, 10 wt%) composites after three-point flexural test: (**a**) AA7075-3wt%B_4_C (1 min 15 s), (**b**) AA7075-5wt%B_4_C (3 min 15 s), (**c**) AA7075-10 wt%B_4_C (4 min 15 s), (**d**) AA7075-3wt%B_4_C (10 min), (**e**) AA7075-5wt%B_4_C (10 min), (**f**) AA7075-10 wt%B_4_C (10 min).

The bending test always ended with fracturing of the sample, which proved the brittleness of the tested materials. Figure 9 presents the fracture surfaces after the three-point bending test. It can be observed that within the matrix region the fracture had a ductile character. The ductile character of the fracture contributed to the formation of dimples as a result of the joining of micropores during deformation, their rupture, and the destruction of adjacent material [32]. Within the region where B_4_C was present, the fracture had a brittle character. Increasing the content of hard particles promoted brittle fracturing. Notwithstanding, there was a good adhesive bond between the boron carbide and the metal matrix at the interface. The most important criterion to determine the type of cracking in the composite is the relationship between the strength of the reinforcement particles and the strength of the matrix-reinforcement interface [33]. As was mentioned before, introducing hard particles into the soft matrix generates a large stress concentration at the phase interface. In addition, as a result of the spark discharge, oxides evaporate from the surface of the particles, which leads to the exposure of pure metallic surfaces in addition to activation and intensification of diffusion phenomena. However, if the matrix–reinforcement bonding is poor, decohesion at the interface will occur before the fracture starts [34]. Additionally, the agglomerations of the reinforcement phase might become potential regions for crack initiation.

## 4. Conclusions

AA7075-*x*B_4_C (*x* = 3, 5, 10 wt%) composites were produced using the spark plasma sintering technique. The density, microstructure, Brinell hardness, and flexural strength of the composites were investigated. The following conclusions can be drawn.

1. The relative density of the sintered composites reached almost 97% and remained at the same level for all the variants of strengthening phase weight fractions and sintering conditions;
2. The sintered composites were characterized by a fine-grained microstructure with a strengthening phase located at the grain boundaries. As a result of the SPS process and cooling, fine precipitates of intermetallic phases from the matrix material were observed in the microstructure. The chemical composition analysis in the micro-areas indicated the presence of phases rich in Mg, Cu and Zn in the microstructure;
3. The XRD analysis of the studied composite material indicated the presence of MgZn_2_, CuZn_2_ and Al_2_CuMg phases in the microstructure. The application of SPS to consolidate the AA7075-B_4_C composites allowed unfavorable solid-state reactions to be avoided between the aluminum and B_4_C particles;
4. With the content of B_4_C particles in the aluminum alloy matrix, the Brinell hardness increased and the bending strength decreased. Extending the holding time resulted in a slight rise in hardness and flexural strength. The exception was the composite with the highest reinforcement content of B_4_C particles (10 wt%), in which the mentioned values were higher for a shorter holding time;
5. The character of the fracture surfaces obtained after the three-point bending test of the examined composite material was ductile within the matrix region and brittle in the region where the reinforcement particles were present.

## Data Availability

Not applicable.
